# Investigation prior to thyroglossal duct cyst excision

**DOI:** 10.1308/003588412X13373405386259

**Published:** 2012-09

**Authors:** Andrew JA Holland

**Affiliations:** Sydney Medical School, The University of Sydney, New South Wales,Australia

## Abstract

Joseph J, Lim K, Ramsden J. Investigation prior to thyroglossal duct cyst. *Ann R Coll Surg Engl* 2012; **94**: 181–184

I enjoyed reading the authors’ report of a computerised questionnaire evaluation of the approach of ear, nose and throat surgeons to the pre-operative assessment of a patient with a clinically diagnosed thyroglossal duct cyst (TDC). With a response rate of 64%, the majority (95%) of the surgeons contacted would arrange an ultrasound (US) scan of the neck, with a moderate-sized minority (32%) requesting thyroid function tests (TFTs). While advocating the value and cost-effectiveness of TFTs to exclude an ectopic thyroid, the authors state in their discussion that ‘Neck ultrasonography can accurately identify normal thyroid tissue in the presence of TDC’.

Unfortunately this statement is not correct. In cases in which the thyroid has not completely descended, the subsequent configuration of the infrahyoid muscles may mimic the US appearance of a normally descended thyroid gland.^1^ While this error may be avoided by high-resolution parasagittal imaging performed by an experienced sonologist with knowledge of the clinical context, this may not always pertain, with radiological attention perhaps diverted by the presumed TDC.^1^

Although I would not disagree with the authors’ conclusions in relation to the results of their questionnaire study, readers should at least be aware of the possibility of an erroneous US report, suggesting the presence of a normally located thyroid gland when this is not the case.
